# Application of a High-Throughput Amplicon Sequencing Method to Chart the Bacterial Communities that Are Associated with European Fermented Meats from Different Origins

**DOI:** 10.3390/foods9091247

**Published:** 2020-09-07

**Authors:** Emiel Van Reckem, Christina Charmpi, David Van der Veken, Wim Borremans, Luc De Vuyst, Stefan Weckx, Frédéric Leroy

**Affiliations:** Research Group of Industrial Microbiology and Food Biotechnology (IMDO), Faculty of Sciences and Bioengineering Sciences, Vrije Universiteit Brussel, Pleinlaan 2, B-1050 Brussels, Belgium; Emiel.Van.Reckem@vub.be (E.V.R.); Christina.Charmpi@vub.be (C.C.); David.Van.Der.Veken@vub.be (D.V.d.V.); Wim.Borremans@vub.be (W.B.); Luc.De.Vuyst@vub.be (L.D.V.); Stefan.Weckx@vub.be (S.W.)

**Keywords:** staphylococci, lactic acid bacteria, fermented meats, high-throughput sequencing, microbiota

## Abstract

Insight into the microbial species diversity of fermented meats is not only paramount to gain control over quality development, but also to better understand the link with processing technology and geographical origin. To study the composition of the microbial communities, the use of culture-independent methods is increasingly popular but often still suffers from drawbacks, such as a limited taxonomic resolution. This study aimed to apply a previously developed high-throughput amplicon sequencing (HTS) method targeting the 16S rRNA and *tuf* genes to characterize the bacterial communities in European fermented meats in greater detail. The data obtained broadened the view on the microbial communities that were associated with the various products examined, revealing the presence of previously underreported subdominant species. Moreover, the composition of these communities could be linked to the specificities of individual products, in particular pH, salt content, and geographical origin. In contrast, no clear links were found between the volatile organic compound profiles of the different products and the country of origin, distinct processing conditions, or microbial communities. Future application of the HTS method offers the potential to further unravel complex microbial communities in fermented meats, as well as to assess the impact of different processing conditions on microbial consortia.

## 1. Introduction

The geographical origin of fermented foods plays an important role in conferring cultural and gastronomic meanings as well as in influencing certain product characteristics [1,2,3]. Naturally fermented dairy products, for instance, have been part of the culinary heritage of many communities throughout the world, showcasing a rich microbial species diversity that is linked to their origin [4]. Similarly, European fermented meat products often provide explicit references to geography [5], which parallels differences in fermentative microbiota [6]. The fermentative microorganisms consist primarily of lactic acid bacteria (LAB) and coagulase-negative staphylococci (CNS). Although many distinctive regional practices exist, key differences in processing can often be reduced to discrepancies in processing between Northern and Southern Europe [7,8]. North-European fermented meat products are usually subjected to a faster fermentation at higher temperatures, leading to a more acid (pH of 5.0 or lower) product than is the case for South-European variants [9]. They also habitually rely on a smoking step that prevents molding, whereas fermented meats in Southern Europe are commonly typified by desirable molding and extensive maturation [2,8,10,11].

Such distinct differences in processing are known to influence the growth and composition of the predominant LAB and CNS communities [6]. For instance, CNS communities in South-European fermented meats are dominated by *Staphylococcus xylosus* and *Staphylococcus equorum*, whereas in Northern Europe there is typically a much higher prevalence of *Staphylococcus carnosus* [6,8,12,13,14,15]. Furthermore, as most fermented sausages are currently produced on an industrial scale, LAB and CNS communities are also heavily influenced by potential starter culture application, which in the case of LAB mostly consists of *Latilactobacillus sakei* and *Pediococcus pentosaceus* and in the case of CNS is limited to *S. xylosus* and/or *S. carnosus* [16,17,18]. In contrast, in traditional fermented sausages, fermentation occurs spontaneously by microorganisms originating from the meat and production environment [19,20]. Depending on the applied processing conditions, traditional fermented sausages can support a much broader CNS species diversity, with *S. equorum*, *Staphylococcus saprophyticus*, and *S. xylosus* being the most prevalent species [17,21].

Culture-dependent as well as culture-independent identification methods have been used to study the bacterial communities of fermented meats [10,22,23]. The former has several disadvantages, as they are often biased towards certain microorganisms because of the selective media and growth conditions used and their inability to recover microorganisms that are less numerically abundant or in a viable but non-cultivable state [23,24]. The latter generally offers a more complete view of the microbiota present, although methods such as denaturing gradient gel electrophoresis of targeted PCR amplicons (PCR-DGGE) can suffer from several drawbacks, such as being labor-intensive, time-consuming, and offering low resolution [23,25].

Recently, culture-independent high-throughput sequencing (HTS) technologies have been applied to analyze microbial communities in fermented foods, including fermented meats, in greater detail [26,27,28]. For this purpose, several studies have relied on the sequencing of one or more hypervariable region(s) of the 16S rRNA gene [25,29]. However, the 16S rRNA gene cannot be used to distinguish between different species within the *Staphylococcus* genus. It is, therefore, necessary to apply other phylogenetic marker genes, such as the *rpoB* or *tuf* gene, when analyzing diverse staphylococcal communities [30,31,32].

The objective of this study was to assess if a previously developed amplicon-based HTS method targeting the 16S rRNA and *tuf* genes [33] can be successfully applied to chart the bacterial communities in European fermented meats. A second goal was to assess if this also allows one to reveal a relationship between those communities and the country of origin and the applied processing parameters of each product (i.e., pH and salt concentration). Additionally, potential links with the volatile organic compound (VOC) profiles of European fermented meat products were investigated.

## 2. Materials and Methods

### 2.1. Sampling and Experimental Set-Up

A total of 15 fermented pork products was obtained from supermarkets in and around Brussels, Belgium. The commercial products originated from Belgium, Germany, France, Italy, and Spain. Three fermented meat products were selected per country of origin for dedicated HTS analysis. Products originating from Belgium, France, and Germany came from at least two different producers per country and products originating from Spain and Italy came from at least three different producers per country. For each product, the label was analyzed to monitor relevant information, i.e., salt concentration and country of origin, and triplicate measurements of pH and the bacterial loads of presumable LAB, determined using de Man–Rogosa–Sharpe (MRS) agar, and CNS, determined using mannitol–salt–agar (MSA), were obtained as described previously [6].

Most fermented meat products were typical representatives of their geographical region of production with respect to their bacterial loads, acidity, and salt content, and were selected based on the data from an earlier study [6]. For all fermented meat products, except one, albeit on different purchases representing different production batches, the bacterial composition has been established previously using a culture-dependent (GTG)_5_-fingerprinting method [6]. These data were used for comparison with the HTS method applied in the present study.

### 2.2. Extraction of Bacterial Genomic DNA

From each fermented meat product, two samples were taken, representing biological duplicates, to analyze the bacterial composition. Bacterial DNA was extracted by first centrifuging a homogenized mixture of sample and recovery diluent at 900× *g* for 10 min to remove coarse impurities. This recovery diluent consisted of a sterile solution of 0.85% (*m*/*v*) NaCl (VWR International, Darmstadt, Germany) and 0.1% (*m*/*v*) bacteriological peptone (Oxoid, Basingstoke, Hampshire, UK). Thereafter, the resulting suspensions were filtered through a 20-μm average pore-size 50-mL Steriflip filter (Merck, Darmstadt, Germany) to remove remaining contaminants. Cell pellets were then obtained by centrifuging at 4000× *g* at 4 °C for 20 min.

Thereafter, DNA extraction was carried out, using a combination of enzymatic, chemical, and mechanical cell lysis. This was followed by phenol/chloroform/isoamyl alcohol extraction and column purification of the DNA, as described in [34], except that the enzymatic steps using lyticase and Zymolyase were excluded from the protocol as molds or yeasts were not targeted in this study.

### 2.3. PCR Assay and Sequencing

To target the overall bacterial communities, the V4 region of the 16S rRNA gene was amplified using the primer set F515/R806 [35], further referred to as V4, that was extended with an Illumina platform-specific 5′ tag, as described previously [36]. In short, PCR assay conditions encompassed an initial denaturation step at 94 °C for 5 min, succeeded by 35 cycles of denaturation at 94 °C for 45 s, annealing at 50 °C for 60 s, and extension at 72 °C for 90 s, and a final extension step at 72 °C for 10 min. To unravel the staphylococcal communities in more detail, a 379-bp region of the *tuf* gene with sufficient discriminatory power to distinguish different CNS species was amplified using primer set Tuf387/765 (Table 1). This primer set has been designed previously, based on a custom database containing 2556 *tuf* gene sequences, representing 48 staphylococcal species selected from the European Nucleotide Archive of the European Bioinformatics Institute (ENA/EBI), and allows distinguishing staphylococcal communities to species level [33]. The PCR assay conditions comprised an initial denaturation step at 94 °C for 2 min, 25 cycles of denaturation at 94 °C for 30 s, annealing at 55 °C for 60 s, and extension at 72 °C for 3 min, and a final extension at 72 °C for 7 min. Next, the amplicons were purified (Wizard Plus SV gel and PCR clean-up system; Promega, Madison, WI, USA), subjected to size selection (Agencourt AMPure XP PCR purification magnetic beads; Beckman Coulter, Brea, CA, USA), and sequenced (Illumina MiSeq sequencing platform; VUB-ULB BRIGHTcore sequencing facility, Jette, Belgium). A mock community (RM3), containing known ratios of *S. carnosus*, *Staphylococcus epidermidis*, *S. equorum*, *Staphylococcus haemolyticus*, *Staphylococcus hominis*, *Staphylococcus lugdunensis*, *S. saprophyticus*, *Staphylococcus sciuri*, *Staphylococcus succinus*, and *S. xylosus*, was used to check the performance of the PCR assays and subsequent sequencing.

### 2.4. Bioinformatic Analysis

Processing of the V4 amplicons was similar to the method described in Zhang et al. [37], whereby amplicon sequence variants (ASVs) were determined using the DADA2 package (version 1.10.1) [38] and the SILVA database (version 138) [39]. For the amplicons generated with primer set Tuf387/765, taxonomy was assigned using the custom database containing 2556 *tuf* gene sequences as described above. In the case of the *tuf* gene, ASVs were only classified to species level if a 100% match with a sequence in the custom database was found. If taxonomy could not be assigned using the custom database, ASVs with more than 1000 reads were aligned to the non-redundant nucleotide database nt of the National Center for Biotechnological Information (NCBI, Bethesda, MA, USA), using blastn as an alignment tool [40]. ASVs were assumed to be present in a sample if they amounted to more than 0.01% of the total reads.

### 2.5. Volatile Organic Compound Profiling

Non-targeted VOC profiling was conducted in triplicate by headspace/solid-phase microextraction coupled to gas chromatography and time-of-flight mass spectrometry (HS/SPME-GC-TOF-MS), using a Trace 1300 gas chromatograph (Thermo Fisher, Waltham, MA, USA) equipped with a Stabilwax-MS column (30 m by 0.25 mm, film thickness of 0.25 µm; Restek, Bellefonte, PA, USA) and coupled to a BenchTOF-HD mass spectrometer operating with electron impact ionization at 70 eV (Markes International, Llantrisant, Wales, UK). For analysis, approximately 10 g of the fermented meat product sample was frozen using liquid nitrogen (Airliquide, Paris, France) and grounded into a fine powder with a coffee grinder (DeLongi KG49, Treviso, Italy). Subsequently, 1.5 g of grounded powder was incubated in a 10-mL screw-top headspace vial at 60 °C for 10 min, after which the sample was exposed to an SPME fiber (DVB/CAR/PDMS; Sigma-Aldrich, St. Louis, MO, USA) at 60 °C for 20 min. To each sample, 10 µL of a 10 ppm toluene-D8 solution (Sigma-Aldrich) was added as an internal standard. The VOCs adsorbed to the SPME fiber were thermally desorbed at 250 °C with a split rate of 50 mL/min. The GC oven temperature program consisted of an initial step at 40 °C for 1.5 min, followed by a continuous increase to 225 °C at 10 °C/min, and finally, the temperature was kept constant at 225 °C for 15 min. Helium gas (Praxair, Danbury, CT, USA) was used as the carrier gas at a flow rate of 1 mL/min. The TOF-MS was scanned in the *m/z* range of 35 to 400 after 2 min. The raw data were deconvoluted by TOF-DS software (Markes). Afterward, peaks were identified based on the NIST library (National Institute of Standard and Technology, Gaitherburg, MD, USA) and the use of commercially available standards [37,41]. For each identified compound, the peak area was normalized to the peak area of the internal standard.

### 2.6. Statistics

RStudio software (version 3.5.2, Rstudio, Boston, USA) was used for all statistical analyses and tests [42].

Assessment of inter-sample diversity (beta-diversity) was achieved by performing a permutational multivariate analysis of variance (PERMANOVA) based on Bray–Curtis dissimilarity scores to ascertain differences in bacterial composition between the fermented meat products investigated. Subsequently, a series of pairwise PERMANOVA comparisons and a similarity percentage analysis (SIMPER) were performed to assess the differences in bacterial communities between fermented meat products produced in the countries mentioned above. The vegan (version 2.5-4) [43] and RVAideMemoire packages (version 0.9-73) [44] were applied for this purpose.

Heatmaps of the non-targeted VOC compound profiling were calculated and clustered using the packages ComplexHeatmap (version 2.0.0) [45] and circlize (version 0.4.7) [46]. Hierarchical clustering was performed according to the average distance between the points in the clusters, based on a correlation matrix of the entire data set. PERMANOVA was used to assess differences in VOC profiles according to the country of origin and/or the prevailing microbial communities, based on Bray–Curtis dissimilarity scores.

## 3. Results

### 3.1. Bacterial Communities in Fermented Meat Products from Different Origins

The characteristics with respect to the pH, salt content, and bacterial loads of each sample of the commercial fermented meat products examined are provided in Table 2. Most products were typical representatives of their geographical region of production. Only the Belgian fermented meat product encoded BE3, which claimed to be produced “à l’ancienne” (i.e., according to “tradition”), was somewhat atypical, as it had a considerably higher pH (5.7) than the other Belgian products with a pH of 5.1 (BE1) and 5.0 (BE2) (Table 2).

**Figure 1:**
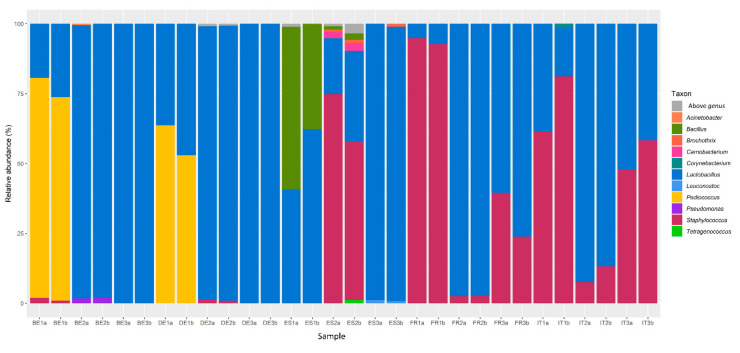
Distribution of amplicon sequencing variants (ASVs) of the V4 region of the 16S rRNA gene in selected commercial fermented meat products originating from BE (Belgium), DE (Germany), ES (Spain), FR (France), and IT (Italy), with “a” and “b” annotations in the sample names representing biological duplicate samples from the same product.

In addition, the bacterial communities present in the various fermented meat products were assessed through amplicon-based HTS, based on 83,642 and 71,971 raw V4 and Tuf387/765 sequence reads on average per sample, respectively. The V4 sequence reads provided a first overview of the bacterial genera present in the different fermented meat products (Figure 1). It unveiled that *Lactobacillus* was present in all samples, no matter the geographical origin of the product. In contrast, *Pediococcus* was only found in samples from Belgian and German fermented meat products and *Pseudomonas* was exclusive to one sample of a Belgian product. Samples from Spanish fermented meat products showcased the greatest microbial diversity, with genera such as *Bacillus*, *Brochothrix*, *Carnobacterium*, *Corynebacterium*, *Leuconostoc*, and *Tetragenococcus* being present. *Staphylococcus* was only found sporadically via this approach in Belgian, German, and Spanish fermented meat products, whereas it was present in all French and Italian ones.

**Figure 2:**
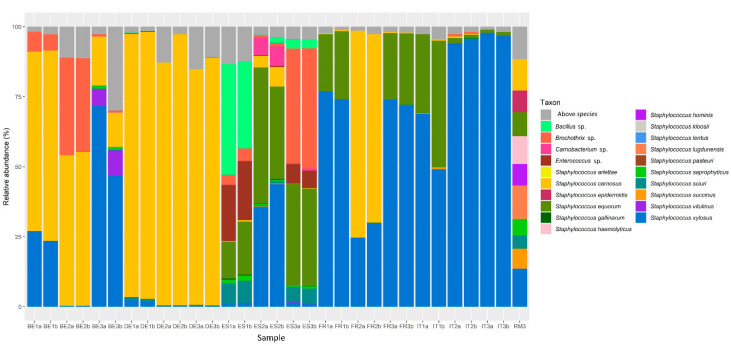
Distribution of amplicon sequencing variants (ASVs) of a partial region of the *tuf* gene in selected commercial fermented meat products originating from BE (Belgium), DE (Germany), ES (Spain), FR (France), and IT (Italy), with “a” and “b” annotations in the sample names representing biological duplicate samples from the same product. RM3 represents the mock community used to check the performance of the PCR assay and subsequent sequencing.

The Tuf387/765 sequence reads obtained by partial *tuf* gene sequencing gave a more detailed view of the CNS communities present in the different fermented meat products (Figure 2). All species present in the mock community (RM3) were correctly identified. Sequences corresponding with several non-*Staphylococcus* genera were also encountered, namely *Bacillus*, *Brochothrix*, *Carnobacterium*, and *Enterococcus. Brochothrix* was encountered in Belgian, German, Italian, and Spanish fermented meat products. *Bacillus*, *Carnobacterium*, and *Enterococcus* were only found in Spanish and German fermented meat products, although in the latter only a small number of reads (<0.3%) could be ascribed to these genera.

**Figure 3:**
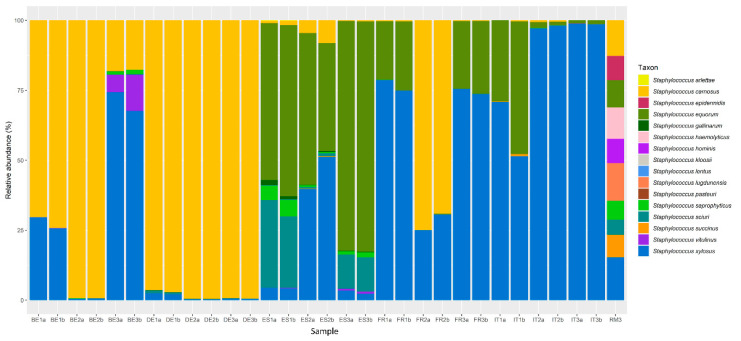
Distribution of amplicon sequencing variants (ASVs) of a partial region of the *tuf* gene assigned to staphylococcal species in selected commercial fermented meat products originating from BE (Belgium), DE (Germany), ES (Spain), FR (France), and IT (Italy), with “a” and “b” annotations in the sample names representing biological duplicate samples from the same product. RM3 represents the mock community used to check the performance of the PCR assay and subsequent sequencing.

Taking into account the only sequence reads attributed to *Staphylococcus* species using partial *tuf* gene sequencing, differences were found between the CNS communities of the fermented meat products from different countries (Figure 3). In Belgian fermented meat products, most sequence reads were assigned to *S. carnosus*, followed by *S. xylosus.* Additionally, *Staphylococcus pasteuri*, *S. sciuri*, *S. saprophyticus*, and *Staphylococcus vitulinus* were found, albeit mostly in minor relative abundances. German fermented meat products were characterized by a large relative abundance of reads originating from *S. carnosus*, with *S. equorum*, *Staphylococcus gallinarum*, *S. sciuri*, and *S. xylosus* occurring as minor fractions. CNS communities in Spanish fermented meat products exhibited the greatest species diversity, including *Staphylococcus arlettae*, *S. carnosus*, *S. equorum*, *S. gallinarum*, *Staphylococcus lentus*, *S. saprophyticus*, *S. sciuri*, *S. vitulinus*, and *S. xylosus*. In French and Italian fermented meat products, the majority of sequence reads was related to *S. xylosus*, followed by *S. equorum*, *S. carnosus*, *S. saprophyticus*, *S. vitulinus*, *S. succinus*, *S. gallinarum*, *Staphylococcus kloosii*, and *S. sciuri.*

To highlight the greater resolution achieved when studying CNS communities by applying the aforementioned amplicon-based HTS method, the CNS species found in the present study were compared to those previously found culture-dependently in the same products (Table 3) [6].

A PERMANOVA indicated that the country of origin had a significant (*p* < 0.05) impact on the composition of the microbial communities. Pairwise PERMANOVA tests showed that microbial communities from Spanish fermented meat products were significantly different from those found in fermented meat products from all other countries. German fermented meat products had different (*p* < 0.05) microbial profiles than French, Italian, and Spanish variants. The microbial communities of Belgian fermented meat products were not significantly different from their German or French counterparts, whereas French fermented meat products had comparable microbial profiles to the Italian ones. SIMPER highlighted that differences in microbial communities were primarily due to the presence of *S. equorum* in Spanish fermented meat products, *S. carnosus* in Belgian and German ones, and *S. xylosus* in French and Italian fermented meat products (Tables S1–S10).

### 3.2. Volatile Organic Compound Profiling

A total of 186 VOCs were detected across the different European fermented meat products examined, of which 25% were terpenes (e.g., limonene, careen, and pinene), 16% were aromatic hydrocarbons (e.g., 3-methylphenol and eugenol), 10% were esters (e.g., methyl butanoate and methyl octanoate), 9% were alcohols (e.g., 2,3-butanediol and 1-octen-3-ol), and 9% were ketones (e.g., acetoin). Minor VOCs consisted of aldehydes (e.g., 3-methylbutanal, hexanal, and nonanal), alkanes (e.g., octane and decane), carboxylic acids (e.g., acetic acid and 2-methylbutanoic acid), furans (e.g., furfural), sulphuric compounds (e.g., diallyl sulphide), and nitrogen compounds (e.g., 2,6-dimethylpyrazine) (Figure S1). Despite individual products displaying differences in their VOC profiles, no VOC profiles or specific VOCs could be linked to either the country of origin or the prevailing microbial communities of the products or to distinct processing conditions using PERMANOVA.

## 4. Discussion

Due to variations in ingredients and processing technologies, the geographical origin of fermented foods can have a profound impact on their technological properties and microbial diversity [3,4]. This is also true for fermented meat products, where clear-cut differences have for instance been found in the characteristics of Northern and Southern European variants [5,6].

The differences in technological properties, namely salt content and pH, of fermented meat products in the present study are in line with the findings of an earlier study, in which the same fermented meat products were investigated, albeit at another moment in time, thus representing other batches [6]. Generally, the salt content of European cured meat products differs within and across different countries [6,47]. However, these differences are not expected to largely influence the technologically important microbiota, consisting mainly of LAB and CNS, as these microorganisms are commonly well adapted to high-salt levels [21]. The usual contrast in acidification extent between Northern and Southern European fermented meat products was illustrated by the differences in pH between German and Belgian commercial products, on the one hand, and French, Italian, and Spanish ones, on the other hand [6,9,11].

With respect to the microbial diversity, application of amplicon-based HTS methods allowed studying the microbial communities with greater resolution compared to previous culture-dependent studies [6,25]. Sequencing of the V4 hypervariable region of the bacterial 16S rRNA gene showed that the genus *Lactobacillus* was omnipresent in fermented meat products across all producing countries examined. Although the genus *Lactobacillus* has recently been reclassified into 25 genera, of which *Latilactobacillus* and *Lactiplantibacillus* contain the most relevant species for meat fermentation [48], the SILVA database used in the present study was not yet updated at the moment the bioinformatics analysis was performed, hence still *Lactobacillus* is reported here. The presence of *Lactobacillaceae* is not surprising as they are frequently encountered as one of the most important genera in fermented sausages, with multiple species such as *Latilactobacillus sakei* (formerly known as *Lactobacillus sakei*), showing great adaptation to the meat matrix [21,25,49]. The presence of *Pediococcus* in some Belgian and German fermented meat products might be a consequence of its addition as a starter culture, as pediococci, such as *P. pentosaceus*, are sometimes added in Northern Europe and Northern America to hasten acidification under higher fermentation temperatures [6,50]. Spanish fermented meat products were remarkable because of the presence of several uncommon genera, such as *Bacillus*, *Brochothrix*, *Carnobacterium*, *Corynebacterium*, and *Tetragenococcus*. These species have nonetheless all been encountered previously to some degree in the natural microbiota of fermented meat products [51,52,53,54,55].

The fact that the genus *Staphylococcus* was not encountered across all Belgian and German fermented meat product samples when targeting the V4 region, did not necessarily infer its absence, as differences in biomass and 16S rRNA copy numbers may cause less abundant genera to be under the detection limit [56,57]. Indeed, whenever a substantial number of reads were ascribed to *Staphylococcus*, the MSA and MRS counts were comparable, with less than 1.0 log (cfu/g) of difference. Furthermore, high-throughput amplicon sequencing of a part of the *tuf* gene revealed diversified CNS communities in all fermented meat products examined. The CNS communities in French and Italian fermented meat products demonstrated a high relative abundance of *S. xylosus*, which was almost always accompanied by *S. equorum.* Other CNS species, such as *S. carnosus* and *S. saprophyticus*, were encountered intermittently and mostly in low relative abundances. This was in line with previous studies, where *S. xylosus* and *S. equorum* were frequently found to characterize Southern-European fermented meat products because they preferred relatively high pH and low fermentation temperatures [6,10,14,54,58,59]. Spanish fermented meat products displayed a high relative abundance of *S. equorum*, but exhibited a greater CNS species diversity. The latter was largely due to the presence of *S. xylosus*, *S. saprophyticus*, *S. sciuri*, *S. gallinarum*, *S. carnosus*, and *S. vitulinus*, which have also previously been found in spontaneously fermented Southern-European fermented meat products [14,60,61,62,63].

When compared to French and Italian fermented meat products, the occurrence of less common genera and species and the overall lower bacterial loads in Spanish products suggest differences on the level of starter culture application, either because the process is based on spontaneous fermentation or because of poor adaptation of the starter culture bacteria involved to the processing conditions [6,16,64]. In contrast, the relatively high abundance of *S. carnosus* in Belgian and German fermented meat products does indicate starter culture use, as this species is not common in the natural microbiota of spontaneously fermented meat products but routinely used as a starter culture in Northern Europe [8,54,65,66]. Noteworthy in that respect was the case of the Belgian product BE3, which was characterized by a somewhat atypical higher pH of 5.7 for its region of production. Although *S. carnosus* was still found, the relative abundance of *S. xylosus* increased and *S. saprophyticus* and *S. vitulinus* also emerged, all of which have been encountered in previous studies in spontaneously fermented meat products of similar acidity [14,67,68]. As fermented meat products, BE1 and BE3 were manufactured by the same producer, it is likely that the same starter culture was used but that different communities were obtained, due to differences in the processing conditions, although details about the latter are not known. This pronounced difference in the composition of the CNS communities underlines the influence of the pH as a major processing factor, as well as the influence of the processing conditions on the structure of the CNS communities in meat fermentation, whether or not starter cultures are used [7,15,64].

Compared to the culture-dependent methodology applied previously on the same commercial fermented meat products [6], amplicon-based HTS allowed for the charting of the CNS communities with far greater resolution (Table 3). Whereas the dominant CNS species were comparable, amplicon-based HTS allowed for the uncovering of several subdominant CNS species. Failure of detection of the latter by culture-dependent methods was due to limitations imposed by the cultivation step and the subsequent work needed to obtain a sufficient number of isolates, fingerprints, and sequencing data. This showcases the capability of amplicon-based HTS methods to rapidly unravel complex microbial communities in fermented foods in a satisfactory degree of detail [25].

Several VOCs were detected in the fermented meat products examined, which could either be related to microbial metabolism (e.g., acetoin, acetic acid, and 3-methylbutanal), the addition of certain spices and herbs (e.g., terpenes and sulfur compounds), or the processing applied (e.g., the presence of phenolic compounds due to smoking) [41,69,70,71,72,73]. However, no clear trends relating VOC compositions to the presence of certain microbial groups or unique processing practices could be distinguished. For this purpose, a more quantitative approach targeting specific VOCs may be needed.

## 5. Conclusions

The present study demonstrated that an amplicon-based HTS method targeting regions of the 16S rRNA and *tuf* genes allows for an improved exploration of the species diversity of the microbial communities in fermented meats, in particular the staphylococcal communities. Amplicon-based HTS broadened the view on the microbial communities to also encompass several subdominant CNS species that previously may have been underreported. Furthermore, it permitted to emphasize the influence of the processing conditions on the bacterial diversity, indicative of pH and starter culture impact. In the present study, only bacterial communities were targeted using partial 16S rRNA and *tuf* gene sequencing. In the future it might be valuable to target other marker genes as well, to obtain a higher resolution image of the microbial communities, including an overview of yeast species diversity. Future applications of the aforementioned amplicon-based HTS methods offer great potential to further unravel complex microbial communities in fermented meat products and other fermented foods as well as to assess the impact of different processing conditions on the entirety of the microbial consortia present.

## Figures and Tables

**Table 1:** Sequence and amplicon size of the Tuf387/765 primer set.

Primer	Primer Sequence	Amplicon Size (bp)
Tuf387	5’-YCCAATGCCWCAAACKCGTGA-3’	379
Tuf765	5’-RAYTTGHCCACGTTCAACAC-3’	

**Table 2:** Individual and average bacterial counts, pH, and salt content with standard deviation (SD) of selected commercial fermented meat products originating from BE (Belgium), DE (Germany), ES (Spain), FR (France), and IT (Italy). MSA, mannitol-salt-agar (representing presumptive staphylococcal species); MRS, de Man–Rogosa–Sharpe (representing presumptive lactic acid bacterial species).

Sample	MSA Counts [log(CFU/g)]			MRS Agar Counts [log(CFU/g)]			pH			Salt Content [g/100g]		
	Individual	Average	SD	Individual	Average	SD	Individual	Average	SD	Individual	Average	SD
BE1	5.40			6.75			5.12			4.10		
BE2	5.13	5.18	0.20	8.27	7.76	0.87	5.00	5.27	0.37	4.60	4.30	0.26
BE3	5.02			8.25			5.70			4.20		
DE1	5.57			7.14			4.78			3.80		
DE2	5.00	5.17	0.35	7.91	7.64	0.43	4.57	4.66	0.11	3.25	3.43	0.32
DE3	4.94			7.88			4.63			3.25		
ES1	5.72			6.53			5.82			3.00		
ES2	5.80	5.83	0.12	5.60	6.83	1.41	6.04	5.85	0.18	3.00	3.27	0.46
ES3	5.95			8.37			5.69			3.80		
FR1	8.16			7.70			5.70			5.70		
FR2	6.52	7.31	0.82	8.26	8.16	0.42	5.51	5.59	0.10	4.60	4.83	0.78
FR3	7.26			8.52			5.56			4.20		
IT1	8.63			8.44			5.66			3.40		
IT2	6.68	7.34	1.12	8.56	8.34	0.30	5.26	5.57	0.28	3.30	3.67	0.55
IT3	6.70			8.00			5.80			4.30		

**Table 3:** Comparison of the *Staphylococcus* (*S.)* species diversity in selected commercial fermented meat products obtained using culture-dependent methods (data obtained from Van Reckem et al., 2019) *versus* the use of the amplicon-based high-throughput sequencing (HTS) method targeting the *tuf* gene (present study). Fermented meat products originated from BE (Belgium), DE (Germany), ES (Spain), FR (France), and IT (Italy).

Sample	Culture-Dependent Method (Total Number of Isolates)	Amplicon-Based HTS Method
BE1	*S. carnosus* (18)	*S. carnosus, S. vitulinus, S. xylosus*
BE2	*S. carnosus* (29)	*S. carnosus, S. saprophyticus, S. xylosus*
BE3	n.a. ^1^	*S. carnosus, S. pasteuri, S. saprophyticus, S. sciuri, S. xylosus*
DE1	*S. carnosus, S. xylosus* (13)	*S. carnosus, S. gallinarum, S. sciuri, S. xylosus*
DE2	*S. carnosus* (20)	*S. carnosus, S. equorum, S. xylosus*
DE3	*S. carnosus* (15)	*S. carnosus, S. xylosus*
ES1	*S. equorum, S. saprophyticus* (21)	*S. arlettae, S. carnosus, S. equorum, S. gallinarum, S. lentus, S. saprophyticus, S. sciuri, S. xylosus*
ES2	*S. carnosus, S. equorum, S. xylosus* (15)	*S. carnosus, S. equorum, S. gallinarum, S. saprophyticus, S. sciuri, S. succinus, S. xylosus*
ES3	*S. equorum* (14)	*S. carnosus, S. equorum, S. gallinarum, S. saprophyticus, S. sciuri, S. vitulinus, S. xylosus*
FR1	*S. equorum, S. xylosus* (23)	*S. carnosus, S. equorum, S. saprophyticus, S. xylosus*
FR2	*S. equorum, S. xylosus* (13)	*S. carnosus, S. vitulinus, S. xylosus*
FR3	*S. equorum, S. xylosus* (10)	*S. carnosus, S. equorum, S. saprophyticus, S. xylosus*
IT1	*S. equorum, S. xylosus* (12)	*S. equorum, S. succinus, S. xylosus*
IT2	*S. xylosus* (13)	*S. carnosus, S. equorum, S. gallinarum, S. kloosii, S. saprophyticus, S. sciuri, S. xylosus*
IT3	*S. xylosus* (20)	*S. equorum, S. saprophyticus, S. vitulinus, S. xylosus*

^1^ data not available.

## Data Availability

The amplicon sequencing data sets were submitted to the European Nucleotide Archive of the European Bioinformatics Institute (ENA/EBI) and are accessible under the study accession number PRJEB39544 (http://www.ebi.ac.uk/ena/data/view/ PRJEB39544).

## Supplementary Materials

The following are available online at https://www.mdpi.com/2304-8158/9/9/1247/s1, Table S1: Similarity percentage analysis (SIMPER) for staphylococcal species identified in Belgian and German fermented meat products, their contribution (in%) to within-group similarity per country, and the cumulative total (in%) of the contributions, Table S2: Similarity percentage analysis (SIMPER) for staphylococcal species identified in Belgian and Spanish fermented meat products, their contribution (in%) to within-group similarity per country, and the cumulative total (in%) of the contributions, Table S3: Similarity percentage analysis (SIMPER) for staphylococcal species identified in Belgian and French fermented meat products, their contribution (in%) to within-group similarity per country, and the cumulative total (in%) of the contributions, Table S4: Similarity percentage analysis (SIMPER) for staphylococcal species identified in Belgian and Italian fermented meat products, their contribution (in%) to within-group similarity per country, and the cumulative total (in%) of the contributions, Table S5: Similarity percentage analysis (SIMPER) for staphylococcal species identified in German and Spanish fermented meat products, their contribution (in%) to within-group similarity per country, and the cumulative total (in%) of the contributions, Table S6: Similarity percentage analysis (SIMPER) for staphylococcal species identified in German and French fermented meat products, their contribution (in%) to within-group similarity per country, and the cumulative total (in%) of the contributions, Table S7: Similarity percentage analysis (SIMPER) for staphylococcal species identified in German and Italian fermented meat products, their contribution (in%) to within-group similarity per country, and the cumulative total (in%) of the contributions, Table S8: Similarity percentage analysis (SIMPER) for staphylococcal species identified in Spanish and French fermented meat products, their contribution (in%) to within-group similarity per country, and the cumulative total (in%) of the contributions, Table S9: Similarity percentage analysis (SIMPER) for staphylococcal species identified in Spanish and Italian fermented meat products, their contribution (in%) to within-group similarity per country, and the cumulative total (in%) of the contributions, Table S10: Similarity percentage analysis (SIMPER) for staphylococcal species identified in French and Italian fermented meat products, their contribution (in%) to within-group similarity per country, and the cumulative total (in%) of the contributions, Figure S1: Hierarchical clustering analysis and heatmap visualization of semi-quantitative volatile organic compound profiles in fermented meat products originating from BE (Belgium), DE (Germany), ES (Spain), FR (France), and IT (Italy), determined using HS/SPME-GC-TOF-MS.
