# Supplementary material for: Application of a High-Throughput Amplicon Sequencing Method to Chart the Bacterial Communities that Are Associated with European Fermented Meats from Different Origins
Source: Foods. 2020 Sep 7;9(9):1247. doi: 10.3390/foods9091247 (PMC7555677; doi:10.3390/foods9091247)

Table S1. Similarity percentage analysis (SIMPER) for staphylococcal species identified in Belgian and German fermented meat products, their contribution (in %) to within-group similarity per country, and the cumulative total (in %) of the contributions.

| Species | Average | Contribution to Belgian products | Contribution to German products | Cumulative  total |
| --- | --- | --- | --- | --- |
| *Staphylococcus carnosus* | 0.179600 | 63.2 | 98.5 | 0.50 |
| *Staphylococcus xylosus* | 0.161900 | 33.1 | 1.04 | 0.94 |
| *Staphylococcus vitulinus* | 0.016080 | 3.22 | 0.00 | 0.99 |
| *Staphylococcus saprophyticus* | 0.002480 | 0.50 | 0.00 | 0.99 |
| *Staphylococcus sciuri* | 0.001574 | 0.06 | 0.30 | 1.00 |
| *Staphylococcus equorum* | 0.000418 | 0.00 | 0.08 | 1.00 |
| *Staphylococcus gallinarum* | 0.000135 | 0.00 | 0.03 | 1.00 |
| *Staphylococcus succinus* | 0.000014 | 0.00 | 0.00 | 1.00 |
| *Staphylococcus pasteuri* | 0.000013 | 0.00 | 0.00 | 1.00 |
| *Staphylococcus aureus* | 0.000008 | 0.00 | 0.00 | 1.00 |
| *Staphylococcus kloosii* | 0.000000 | 0.00 | 0.00 | 1.00 |
| *Staphylococcus lentus* | 0.000000 | 0.00 | 0.00 | 1.00 |

Table S2. Similarity percentage analysis (SIMPER) for staphylococcal species identified in Belgian and Spanish fermented meat products, their contribution (in %) to within-group similarity per country, and the cumulative total (in %) of the contributions.

| Species | Average | Contribution to Belgian products | Contribution to Spanish products | Cumulative total |
| --- | --- | --- | --- | --- |
| *Staphylococcus equorum* | 0.312200 | 0.00 | 62.4 | 0.36 |
| *Staphylococcus carnosus* | 0.303000 | 63.2 | 2.60 | 0.71 |
| *Staphylococcus xylosus* | 0.154000 | 33.1 | 17.6 | 0.88 |
| *Staphylococcus sciuri* | 0.068730 | 0.06 | 13.8 | 0.96 |
| *Staphylococcus vitulinus* | 0.016420 | 3.22 | 0.25 | 0.98 |
| *Staphylococcus saprophyticus* | 0.011240 | 0.50 | 2.57 | 1.00 |
| *Staphylococcus gallinarum* | 0.003106 | 0.00 | 0.62 | 1.00 |
| *Staphylococcus succinus* | 0.000403 | 0.00 | 0.08 | 1.00 |
| *Staphylococcus lentus* | 0.000269 | 0.00 | 0.05 | 1.00 |
| *Staphylococcus pasteuri* | 0.000013 | 0.00 | 0.00 | 1.00 |
| *Staphylococcus aureus* | 0.000008 | 0.00 | 0.00 | 1.00 |
| *Staphylococcus kloosii* | 0.000000 | 0.00 | 0.00 | 1.00 |

Table S3. Similarity percentage analysis (SIMPER) for staphylococcal species identified in Belgian and French fermented meat products, their contribution (in %) to within-group similarity per country, and the cumulative total (in %) of the contributions.

| Species | Average | Contribution to Belgian products | Contribution to French products | Cumulative total |
| --- | --- | --- | --- | --- |
| *Staphylococcus carnosus* | 0.256400 | 63.2 | 24.2 | 0.48 |
| *Staphylococcus xylosus* | 0.182900 | 33.1 | 59.7 | 0.82 |
| *Staphylococcus equorum* | 0.080180 | 0.00 | 16.0 | 0.97 |
| *Staphylococcus vitulinus* | 0.016060 | 3.22 | 0.01 | 0.99 |
| *Staphylococcus saprophyticus* | 0.002433 | 0.50 | 0.03 | 1.00 |
| *Staphylococcus sciuri* | 0.000280 | 0.06 | 0.00 | 1.00 |
| *Staphylococcus pasteuri* | 0.000013 | 0.00 | 0.00 | 1.00 |
| *Staphylococcus aureus* | 0.000008 | 0.00 | 0.00 | 1.00 |
| *Staphylococcus gallinarum* | 0.000000 | 0.00 | 0.00 | 1.00 |
| *Staphylococcus kloosii* | 0.000000 | 0.00 | 0.00 | 1.00 |
| *Staphylococcus lentus* | 0.000000 | 0.00 | 0.00 | 1.00 |
| *Staphylococcus succinus* | 0.000000 | 0.00 | 0.00 | 1.00 |

Table S4. Similarity percentage analysis (SIMPER) for staphylococcal species identified in Belgian and Italian fermented meat products, their contribution (in %) to within-group similarity per country, and the cumulative total (in %) of the contributions.

| Species | Average | Contribution to Belgian products | Contribution to Italian products | Cumulative total |
| --- | --- | --- | --- | --- |
| *Staphylococcus carnosus* | 0.314600 | 63.2 | 0.25 | 0.46 |
| *Staphylococcus xylosus* | 0.275400 | 33.1 | 85.8 | 0.87 |
| *Staphylococcus equorum* | 0.068490 | 0.00 | 13.7 | 0.97 |
| *Staphylococcus vitulinus* | 0.016050 | 3.22 | 0.03 | 0.99 |
| *Staphylococcus saprophyticus* | 0.002403 | 0.50 | 0.05 | 1.00 |
| *Staphylococcus succinus* | 0.000803 | 0.00 | 0.16 | 1.00 |
| *Staphylococcus sciuri* | 0.000299 | 0.06 | 0.02 | 1.00 |
| *Staphylococcus gallinarum* | 0.000133 | 0.00 | 0.03 | 1.00 |
| *Staphylococcus kloosii* | 0.000041 | 0.00 | 0.01 | 1.00 |
| *Staphylococcus pasteuri* | 0.000013 | 0.00 | 0.00 | 1.00 |
| *Staphylococcus aureus* | 0.000008 | 0.00 | 0.00 | 1.00 |
| *Staphylococcus lentus* | 0.000000 | 0.00 | 0.00 | 1.00 |

Table S5. Similarity percentage analysis (SIMPER) for staphylococcal species identified in German and Spanish fermented meat products, their contribution (in %) to within-group similarity per country, and the cumulative total (in %) of the contributions.

| Species | Average | Contribution to German products | Contribution to Spanish products | Cumulative total |
| --- | --- | --- | --- | --- |
| *Staphylococcus carnosus* | 0.479898 | 98.5 | 2.60 | 0.50 |
| *Staphylococcus equorum* | 0.311756 | 0.08 | 62.4 | 0.82 |
| *Staphylococcus xylosus* | 0.082609 | 1.04 | 17.6 | 0.91 |
| *Staphylococcus sciuri* | 0.067734 | 0.30 | 13.8 | 0.98 |
| *Staphylococcus saprophyticus* | 0.012839 | 0.00 | 2.57 | 0.99 |
| *Staphylococcus gallinarum* | 0.002971 | 0.03 | 0.62 | 1.00 |
| *Staphylococcus vitulinus* | 0.001272 | 0.00 | 0.25 | 1.00 |
| *Staphylococcus succinus* | 0.000408 | 0.00 | 0.08 | 1.00 |
| *Staphylococcus lentus* | 0.000269 | 0.00 | 0.05 | 1.00 |
| *Staphylococcus aureus* | 0.000000 | 0.00 | 0.00 | 1.00 |
| *Staphylococcus kloosii* | 0.000000 | 0.00 | 0.00 | 1.00 |
| *Staphylococcus pasteuri* | 0.000000 | 0.00 | 0.00 | 1.00 |

Table S6. Similarity percentage analysis (SIMPER) for staphylococcal species identified in German and French fermented meat products, their contribution (in %) to within-group similarity per country, and the cumulative total (in %) of the contributions.

| Species | Average | Contribution to German products | Contribution to French products | Cumulative total |
| --- | --- | --- | --- | --- |
| *Staphylococcus carnosus* | 0.371600 | 98.5 | 24.2 | 0.50 |
| *Staphylococcus xylosus* | 0.293300 | 1.04 | 59.7 | 0.89 |
| *Staphylococcus equorum* | 0.079900 | 0.08 | 16.0 | 1.00 |
| *Staphylococcus sciuri* | 0.001523 | 0.30 | 0.00 | 1.00 |
| *Staphylococcus saprophyticus* | 0.000142 | 0.00 | 0.03 | 1.00 |
| *Staphylococcus gallinarum* | 0.000135 | 0.03 | 0.00 | 1.00 |
| *Staphylococcus vitulinus* | 0.000045 | 0.00 | 0.01 | 1.00 |
| *Staphylococcus succinus* | 0.000014 | 0.00 | 0.00 | 1.00 |
| *Staphylococcus aureus* | 0.000000 | 0.00 | 0.00 | 1.00 |
| *Staphylococcus kloosii* | 0.000000 | 0.00 | 0.00 | 1.00 |
| *Staphylococcus lentus* | 0.000000 | 0.00 | 0.00 | 1.00 |
| *Staphylococcus pasteuri* | 0.000000 | 0.00 | 0.00 | 1.00 |

Table S7. Similarity percentage analysis (SIMPER) for staphylococcal species identified in German and Italian fermented meat products, their contribution (in %) to within-group similarity per country, and the cumulative total (in %) of the contributions.

| Species | Average | Contribution to German products | Contribution to Italian products | Cumulative total |
| --- | --- | --- | --- | --- |
| *Staphylococcus carnosus* | 0.491500 | 98.5 | 0.25 | 0.50 |
| *Staphylococcus xylosus* | 0.423600 | 1.04 | 85.8 | 0.93 |
| *Staphylococcus equorum* | 0.068070 | 0.08 | 13.7 | 1.00 |
| *Staphylococcus sciuri* | 0.001518 | 0.30 | 0.02 | 1.00 |
| *Staphylococcus succinus* | 0.000808 | 0.00 | 0.16 | 1.00 |
| *Staphylococcus saprophyticus* | 0.000232 | 0.00 | 0.05 | 1.00 |
| *Staphylococcus gallinarum* | 0.000184 | 0.03 | 0.03 | 1.00 |
| *Staphylococcus vitulinus* | 0.000139 | 0.00 | 0.03 | 1.00 |
| *Staphylococcus kloosii* | 0.000041 | 0.00 | 0.01 | 1.00 |
| *Staphylococcus aureus* | 0.000000 | 0.00 | 0.00 | 1.00 |
| *Staphylococcus lentus* | 0.000000 | 0.00 | 0.00 | 1.00 |
| *Staphylococcus pasteuri* | 0.000000 | 0.00 | 0.00 | 1.00 |

Table S8. Similarity percentage analysis (SIMPER) for staphylococcal species identified in Spanish and French fermented meat products, their contribution (in %) to within-group similarity per country, and the cumulative total (in %) of the contributions.

| Species | Average | Contribution to Spanish products | Contribution to French products | Cumulative total |
| --- | --- | --- | --- | --- |
| *Staphylococcus equorum* | 0.231967 | 62.4 | 16.0 | 0.34 |
| *Staphylococcus xylosus* | 0.230428 | 17.6 | 59.7 | 0.69 |
| *Staphylococcus carnosus* | 0.123472 | 2.60 | 24.2 | 0.87 |
| *Staphylococcus sciuri* | 0.069009 | 13.8 | 0.00 | 0.97 |
| *Staphylococcus saprophyticus* | 0.012698 | 2.57 | 0.03 | 0.99 |
| *Staphylococcus gallinarum* | 0.003106 | 0.62 | 0.00 | 1.00 |
| *Staphylococcus vitulinus* | 0.001272 | 0.25 | 0.01 | 1.00 |
| *Staphylococcus succinus* | 0.000403 | 0.08 | 0.00 | 1.00 |
| *Staphylococcus lentus* | 0.000269 | 0.05 | 0.00 | 1.00 |
| *Staphylococcus aureus* | 0.000000 | 0.00 | 0.00 | 1.00 |
| *Staphylococcus kloosii* | 0.000000 | 0.00 | 0.00 | 1.00 |
| *Staphylococcus pasteuri* | 0.000000 | 0.00 | 0.00 | 1.00 |

Table S9. Similarity percentage analysis (SIMPER) for staphylococcal species identified in Spanish and Italian fermented meat products, their contribution (in %) to within-group similarity per country, and the cumulative total (in %) of the contributions.

| Species | Average | Contribution to Spanish products | Contribution to Italian products | Cumulative total |
| --- | --- | --- | --- | --- |
| *Staphylococcus xylosus* | 0.341200 | 17.6 | 85.8 | 0.50 |
| *Staphylococcus equorum* | 0.246100 | 62.4 | 13.7 | 0.86 |
| *Staphylococcus sciuri* | 0.068930 | 13.8 | 0.02 | 0.96 |
| *Staphylococcus saprophyticus* | 0.012610 | 2.57 | 0.05 | 0.97 |
| *Staphylococcus carnosus* | 0.012040 | 2.60 | 0.25 | 0.99 |
| *Staphylococcus gallinarum* | 0.002973 | 0.62 | 0.03 | 1.00 |
| *Staphylococcus vitulinus* | 0.001272 | 0.25 | 0.03 | 1.00 |
| *Staphylococcus succinus* | 0.000972 | 0.08 | 0.16 | 1.00 |
| *Staphylococcus lentus* | 0.000269 | 0.05 | 0.00 | 1.00 |
| *Staphylococcus kloosii* | 0.000041 | 0.00 | 0.01 | 1.00 |
| *Staphylococcus aureus* | 0.000000 | 0.00 | 0.00 | 1.00 |
| *Staphylococcus pasteuri* | 0.000000 | 0.00 | 0.00 | 1.00 |

Table S10. Similarity percentage analysis (SIMPER) for staphylococcal species identified in French and Italian fermented meat products, their contribution (in %) to within-group similarity per country, and the cumulative total (in %) of the contributions.

| Species | Average | Contribution to French products | Contribution to Italian products | Cumulative total |
| --- | --- | --- | --- | --- |
| *Staphylococcus xylosus* | 0.162700 | 59.7 | 85.8 | 0.44 |
| *Staphylococcus carnosus* | 0.120500 | 24.2 | 0.25 | 0.76 |
| *Staphylococcus equorum* | 0.088500 | 16.0 | 13.7 | 1.00 |
| *Staphylococcus succinus* | 0.000803 | 0.00 | 0.16 | 1.00 |
| *Staphylococcus saprophyticus* | 0.000195 | 0.03 | 0.05 | 1.00 |
| *Staphylococcus vitulinus* | 0.000139 | 0.01 | 0.03 | 1.00 |
| *Staphylococcus gallinarum* | 0.000133 | 0.00 | 0.03 | 1.00 |
| *Staphylococcus sciuri* | 0.000077 | 0.00 | 0.02 | 1.00 |
| *Staphylococcus kloosii* | 0.000041 | 0.00 | 0.01 | 1.00 |
| *Staphylococcus aureus* | 0.000000 | 0.00 | 0.00 | 1.00 |
| *Staphylococcus lentus* | 0.000000 | 0.00 | 0.00 | 1.00 |
| *Staphylococcus pasteuri* | 0.000000 | 0.00 | 0.00 | 1.00 |

Figure S1. Hierarchical clustering analysis and heatmap visualization of semi-quantitative volatile organic compound profiles in fermented meat products originating from BE (Belgium), DE (Germany), ES (Spain), FR (France), and IT (Italy), determined using HS/SPME-GC-TOF-MS.


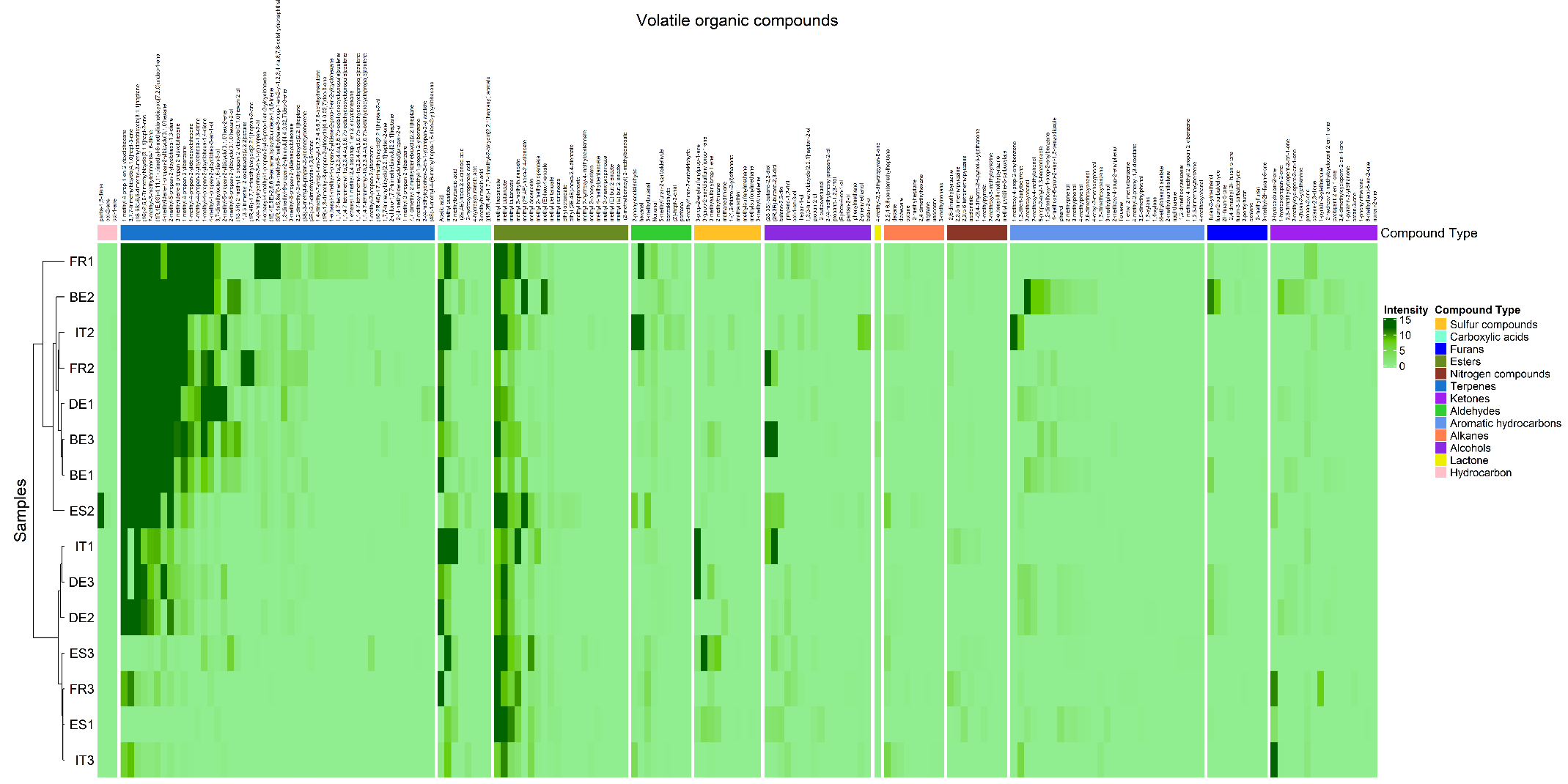

Supplement: Supplementary file 1 [file foods-09-01247-s001.zip › foods-920128-supplementary.docx]
